# Regulatory Readiness for Paper Replacement in Global Pharmaceutical Electronic Labeling: Cross-Sectional Comparative Policy Analysis

**DOI:** 10.2196/96787

**Published:** 2026-08-18

**Authors:** Da Eun Lee, Ji Sun Chun, Mari Kim, In-Wha Kim, Jung Mi Oh

**Affiliations:** 1 College of Pharmacy Seoul National University Seoul Republic of Korea; 2 Research Institute of Pharmaceutical Sciences Seoul National University Seoul, Seoul Republic of Korea; 3 Natural Products Research Institute Seoul National University Seoul Republic of Korea

**Keywords:** digital health, drug labeling, electronic labeling, health information systems, health policy, MeSH, pharmaceutical preparations, policy analysis, regulatory framework

## Abstract

**Background:**

Accurate and up-to-date medicinal product information is essential for the safe and effective use of medicines. Electronic labeling (e-labeling) has emerged as a potential alternative to paper-based package inserts by enabling timely updates, improving accessibility, and supporting integration with digital health systems. However, regulatory approaches to pharmaceutical e-labeling remain fragmented across jurisdictions.

**Objective:**

This study examined how e-labeling policies have developed across International Coalition of Medicines Regulatory Authorities (ICMRA) member jurisdictions and explored the conditions associated with policies that position e-labeling as a replacement for paper-based product information.

**Methods:**

We conducted a cross-sectional comparative policy analysis of publicly available national regulatory documents on pharmaceutical e-labeling issued between January 1, 2012, and May 31, 2026. The analysis focused on how regulatory intent, legal authority, implementation mechanisms, and replacement pathways were articulated in official policy documents across jurisdictions. A main corpus of official jurisdictional policy documents was supplemented by a triangulation corpus of international stakeholder documents. Hybrid thematic analysis and content analysis were used to identify and compare policy themes across jurisdictions. A 4-level e-labeling regulatory maturity framework was developed using 3 indicators: implementation status, level of authority, and expansion toward advanced considerations. Fuzzy-set qualitative comparative analysis (fsQCA) was then conducted across 19 jurisdictions to identify configurational patterns associated with paper-label replacement.

**Results:**

A total of 81 policy documents from 20 jurisdictions were included in the main analysis, and 27 documents were included in the triangulation corpus. Nine themes were identified across 3 domains: e-labeling scope and implementation strategy, operational infrastructure, and advanced considerations. Most policies addressed target product information, medicine categories, transition approaches, interface mechanisms, and responsible entities. In contrast, structured digital formats, interoperability, and personalized information were less consistently addressed. The maturity assessment showed substantial variation across jurisdictions. Brazil and the Netherlands were classified at the highest maturity level, reflecting implementation with a legal basis and expansion toward advanced functions. The fsQCA identified 1 conservative sufficient configuration associated with paper-label replacement: digital infrastructure readiness, high e-labeling policy maturity, and explicit responsibility of the marketing authorization holder.

**Conclusions:**

Pharmaceutical e-labeling has moved beyond early policy discussion in many ICMRA member jurisdictions, but regulatory maturity remains uneven. While most jurisdictions enable electronic access to product information, relatively few have established the legal and operational foundations to replace paper labeling. Paper-label replacement was associated with mature regulatory frameworks and clear operational responsibility. By combining a cross-jurisdictional regulatory maturity framework with fsQCA, this study provides a novel comparative approach that extends beyond previous descriptive studies by identifying the conditions associated with paper-label replacement. The proposed framework may offer a structured approach to assessing regulatory readiness and prioritizing the efforts required for effective legal development and implementation.

## Introduction

The dissemination of accurate and up-to-date medicinal product information is fundamental to the safe and effective use of medicines. Such information is traditionally provided through printed materials, including labels and package inserts (PIs). A “label” typically refers to information printed on the immediate or outer packaging of a medicine, whereas a PI is a more comprehensive document provided inside a medicine’s packaging. Although paper-based PIs have long served as the principal medium for communicating product information, they pose significant challenges, particularly in promptly reflecting updated safety, efficacy, and administration information [1]. Delays in revising and distributing printed materials may impede the timely use of the most current information in clinical practice and by patients. Additional concerns, including the financial and labor costs associated with label revisions, reduced readability due to the increasing volume of required information, and environmental burdens, further underscore the need for more efficient mechanisms to provide medicinal product information [2].

In response to these challenges, electronic labeling (e-labeling) has emerged as a digital health innovation that enables medicinal product information to be provided electronically. e-Labeling is not limited to the digitization of paper-based PIs; rather, it may involve the establishment of regulatory and technical infrastructures for timely updates, user access, information governance, and integration with broader digital health systems [1,2]. By enabling timely updates, improving readability, and enhancing user accessibility, e-labeling can serve as a core component of the broader digital health ecosystem. This system ultimately aims to strengthen patient safety, optimize medication use, and improve the overall efficiency of health care delivery. Aligned with the World Health Organization (WHO)’s Global Strategy on Digital Health, which emphasizes the integration of digital technologies to enhance health system performance, this approach has attracted considerable global interest, with numerous countries currently conducting pilot projects to evaluate the effectiveness and feasibility of e-labeling [3-6].

Despite these advantages, the global landscape of e-labeling remains highly fragmented. There are substantial differences among countries in terms of regulatory definitions, legal frameworks, and implementation strategies [1,2]. These differences matter because the transition from paper-based to electronic product information (ePI) depends on more than the availability of digital formats. It also requires sufficient regulatory authority, implementation capacity, responsibility allocation, and system-level readiness. However, previous studies have examined e-labeling primarily from technological or national policy perspectives [2], and comprehensive qualitative comparative analysis (QCA) of e-labeling policies across multiple jurisdictions remains scarce. As a result, limited evidence is available on how e-labeling policies differ across jurisdictions, how mature these policies are, and what combinations of conditions may support the movement toward paper replacement.

This study aimed to address this gap by examining how pharmaceutical e-labeling policies have evolved across global jurisdictions and what this variation implies for the transition from paper-based to ePI. First, we compared cross-jurisdictional differences in policy scope, implementation, operational infrastructure, and advanced considerations to identify major policy trends and regulatory gaps. Second, we assessed jurisdiction-level policy maturity to locate each jurisdiction within the regulatory development of pharmaceutical e-labeling. Lastly, we explored how contextual and policy-intrinsic conditions combine in jurisdictions where e-labeling is positioned as a replacement for paper-based product information. Accordingly, the study was designed to analyze the regulatory articulation of e-labeling policies across jurisdictions rather than to map scholarly evidence on the topic, with particular attention to regulatory maturity and the conditions associated with paper-label replacement.

## Methods

### Study Design and Data Sources

This cross-sectional comparative study integrated qualitative policy document analysis with a comparative configurational approach. Its aim was to compare how regulatory intent, legal authority, implementation mechanisms, and replacement pathways are articulated in official policy documents across jurisdictions. We used policy document analysis [7] to examine national policies on e-labeling of pharmaceutical products. Document analysis was conducted as an iterative process of skimming, close reading, and interpretation, through hybrid thematic analysis combined with content analysis. Subsequently, fuzzy-set qualitative comparative analysis (fsQCA) [8] was conducted to explore factors associated with the cross-jurisdictional differences identified during document analysis. The Standards for Reporting Qualitative Research (SRQR) checklist was used to report this study [9].

Given the relatively recent emergence of e-labeling policies, we purposively selected jurisdictions represented in the International Coalition of Medicines Regulatory Authorities (ICMRA) as the focus of analysis. The ICMRA is an informal consortium of leaders from national and regional medicine regulatory authorities. Because of its strategic leadership and coordinated actions in addressing global regulatory challenges, the ICMRA was considered an appropriate reference point for identifying jurisdictions at the forefront of regulatory convergence in this area [10].

To enhance the reliability and credibility of the analysis, we constructed 2 document corpora: a main corpus and a triangulation corpus. The main corpus consisted of publicly available national policies or official notices concerning e-labeling of pharmaceutical products. The triangulation corpus consisted of relevant documents issued by international stakeholder organizations, including the WHO, the International Federation of Pharmaceutical Manufacturers and Associations (IFPMA), the European Federation of Pharmaceutical Industries and Associations (EFPIA), the Global Self-Care Federation (GSCF), and the Asia Partnership Conference of Pharmaceutical Associations (APAC-Asia). These organizations were selected because they include stakeholders involved in the regulation, manufacture, distribution, or use of e-labeling across multiple jurisdictions. Additional evidence from the triangulation corpus was used for 3 purposes. First, to determine whether the absence of eligible documents reflected a genuine lack of policy consideration or merely the lack of policy transparency. Second, to verify the implementation status of each jurisdiction. Third, to cross-validate themes identified through coding of the main corpus. The triangulation process was designed to enhance the consistency and robustness of the interpretation of document analysis, by corroborating findings across multiple authoritative sources.

### Search Strategy

We adopted a structured search strategy guided by the PRISMA-S (Preferred Reporting Items for Systematic Reviews and Meta-Analyses Literature Search Extension) [11] to screen documents relevant to the purpose of this study. This strategy enabled us to maintain a rigorous and credible workflow while allowing flexibility to include diverse types of policy documents.

For the main corpus, searches were performed on the official websites of national health ministries, medicines regulatory agencies, and other governmental bodies. Considering the broad variation in terminology used for regulatory systems, medicinal product information, and e-labeling across jurisdictions, we used comprehensive keywords covering policy documents, medicines, product information, and digital delivery formats. Core search terms included “policy,” “law,” “regulation,” “guideline,” and “notification”; “medicinal product,” and “medicine”; “package insert,” “leaflet,” “label,” and “product information”; “electronic,” “mobile,” “e-label,” “Quick Response (QR) code,” and “ePI.” Search queries were adapted for each country or authority according to locally used terminology identified during the search process. For the triangulation corpus, we searched for documents on the websites of predefined international organizations using the same keywords, only excluding policy-related terms.

To ensure methodological rigor and minimize potential selection bias, 2 authors (DEL and JSC) independently performed the search and screening processes. The search was conducted according to a predefined, transparent, and reproducible protocol, with all search terms, sources, and procedures comprehensively documented to enable replication and verification by other researchers. Any discrepancies in document identification were resolved through discussion and consensus.

Detailed source websites, search queries, URLs, and inclusion/exclusion decisions for the main and triangulation corpora are provided in Tables S1 and S2 in Multimedia Appendix 1, in accordance with SRQR principles.

### Eligibility Criteria and Selection

Eligible policy documents were publicly available official documents issued by a government or medicines regulatory authority in 2024 ICMRA member jurisdictions [10] between January 1, 2012, and May 31, 2026. Jurisdictions for which no eligible document was identified were classified as having no publicly accessible documentation within the search framework, rather than as lacking an e-labeling policy.

The publication date range was restricted to reflect contemporary policies while minimizing the inclusion of outdated documents. The early 2010s were selected as the starting point to capture global trends in e-labeling, as this period coincided with the initial implementation of e-labeling–related policies by the US Food and Drug Administration (FDA) and the European Medicines Agency (EMA). Documents written in languages other than English or Korean were analyzed after translation, back-translation, and peer-review process to ensure accuracy of interpretation. These documents included materials written in Japanese, Chinese, Portuguese, Italian, and German.

To ensure relevance to the e-labeling of pharmaceutical products, all documents were manually examined and excluded if they met any of the following criteria: (1) the policy did not apply to pharmaceutical products, (2) the policy did not address e-labeling, or (3) the policy focused solely on administrative aspects of drug labeling, such as distribution tracking or electronic submission for approval. Two authors (DEL and JSC) independently screened and selected the documents based on inclusion and exclusion criteria. Any disagreements were resolved through discussion, with final consensus reached in consultation with a third reviewer.

For the triangulation corpus, we included publicly available documents officially issued by the predefined international organizations that addressed e-labeling. Documents limited to exceptional circumstances, such as COVID-19–specific measures, or documents focused on less directly relevant aspects of e-labeling, such as environmental costs, were excluded after full-text assessment.

### Policy Thematic Analysis

For each included policy document, we first extracted basic document characteristics, including the issuing jurisdiction, issuing authority, date of first publication, date of most recent update, and level of authority. The level of authority was classified into 2 categories: legal and nonlegal. The “legal” category included legally binding instruments enacted and announced for implementation, such as laws, acts, or rules. The “nonlegal” category included guidance, notifications, official reports, pilot project documents, and similar materials that suggest best practices or implementation considerations. In this context, we considered legal instruments to represent a higher level of authority than nonlegal ones.

Policy documents were analyzed using a hybrid thematic analysis combined with content analysis. Matsui et al [2] examined the current status of e-labeling and suggested 5 factors to be considered based on a survey completed by APAC-Asia member associations in November 2021. At that time, discussions on e-labeling were just beginning, the level of adoption varied by country, and consequently, there were no common standards. Nevertheless, examining the current status and the 5 factors made it possible to identify the foundations that needed to be established from the outset to introduce e-labeling, as well as the aspects to which e-labeling could be further expanded. Drawing on prior literature on e-labeling of medicinal products, we identified three key requirements for effective implementation and defined these as analytic domains: (1) e-labeling scope and implementation strategy, (2) operational infrastructure for e-labeling implementation, and (3) advanced considerations for e-labeling.

During the initial phase, we skimmed policy documents to gain an overview of their structure, scope, and regulatory context. Recurring themes were then inductively derived from the documents of the main corpus and subsequently deductively mapped onto the 3 predefined domains. Documents in the triangulation corpus were then coded deductively using the themes identified from the main corpus. Newly recognized patterns related to the regulation and implementation of e-labeling were also coded separately. Two coders (DEL and JSC) independently performed sentence-level coding using NVivo (version 15; QSR International). Cohen κ coefficient was assessed to estimate intercoder agreement. Coding discrepancies were reviewed and resolved through discussion and consensus. The thematic analysis procedure and reporting were reviewed against Braun and Clarke’s [12] 15-point checklist for good thematic analysis to ensure coherence among the analytic approach, coding process, thematic interpretation, and reporting.

After thematic coding, content analysis was conducted to compare how each theme was addressed across jurisdictions. For each theme, coded excerpts were reviewed at the jurisdiction level, with particular attention to the original expressions used in each policy document. Based on this process, jurisdiction-specific policy characteristics were interpreted and summarized using common descriptive terms that enabled cross-jurisdictional comparison while preserving the contextual meaning of each policy document. These summaries were compared within each analytic domain to identify cross-jurisdictional policy trends, distinctive approaches, and regulatory gaps, which were subsequently used to inform the jurisdiction-level maturity assessment.

### Maturity Level Assessment

We assessed the maturity of e-labeling regulation in each jurisdiction using a framework informed by the WHO Global Benchmarking Tool (GBT) [13]. The WHO GBT conceptualizes regulatory maturity as a progression from a fragmented system with foundational regulatory functions, to an evolving system in which basic functions are implemented but oversight remains under development, to a well-functioning and legally established regulatory system, and finally to an advanced system characterized by continuous improvement, international alignment. Drawing on this maturity logic, we developed a 4-level framework tailored to pharmaceutical e-labeling regulation.

In this adapted framework, level 1 represented a fragmented or preimplementation stage, in which e-labeling was absent, proposed, or under development but had not yet been implemented through an identifiable policy pathway. Level 2 represented an evolving regulatory system, in which e-labeling had been implemented or piloted but was primarily supported by legal or nonbinding policy instruments. Level 3 represented a well-established regulatory system, in which e-labeling had been implemented on the basis of a legal, statutory, or otherwise formally binding policy instrument. Level 4 represented an advanced regulatory system, in which legally grounded and implemented e-labeling policies extended beyond basic electronic provision of product information to address broader implementation considerations, such as interoperability, integration with digital health information systems, or the provision of personalized information.

Levels were assigned using 3 dimensions derived from this adapted maturity logic: implementation status, regulatory authority, and policy expansion. Implementation status was assessed first, followed by the legal or binding nature of the policy instrument. Policy expansion was considered only after implementation and formal authority had been established; therefore, advanced technical features alone were not sufficient for assignment to level 4. When evidence was mixed or ambiguous, the lower level was assigned unless both operational implementation and formal authority were clearly documented. Implementation status and regulatory authority were primarily assessed using jurisdictional policy documents in the main corpus. When national policy documents did not clearly specify implementation status, documents in the triangulation corpus were used to cross-validate whether e-labeling had been implemented, remained under development, or was not yet implemented. This framework was used to classify assessable jurisdictions from level 1 to level 4 as an analytic description of e-labeling policy development, rather than as a normative ranking of overall regulatory performance.

### QCA

An fsQCA was conducted to identify configurations of conditions associated with national policies in which e-labeling was positioned as a replacement for paper-based product information. The unit of analysis was the jurisdiction. The outcome was defined as membership in the set of jurisdictions with a paper-replacement policy for e-labeling. This outcome was selected because triangulation with international stakeholder documents showed that the transition from paper-based to ePI was repeatedly framed as a future policy direction, ranging from staged dual-system transition to eventual replacement or exclusive use of electronic leaflets [14-18].

Candidate conditions were identified from jurisdiction-level contextual characteristics and policy-intrinsic characteristics derived from the thematic and content analysis. Several contextual variables, including income category, government digitalization [19] and language variability [20], were initially considered because they could plausibly influence the feasibility or demand of e-labeling. However, preliminary distributional assessment showed limited cross-jurisdictional variation in these variables. To avoid adding conditions that would increase truth-table complexity without contributing meaningful configurational differentiation, they were not included in the final analysis. The final fsQCA model included 5 conditions: digital infrastructure readiness, e-labeling policy scope, e-labeling policy maturity, explicit marketing authorization holders (MAHs) responsibility, and explicit national competent authority (NCA) responsibility. Digital infrastructure readiness was retained as the contextual condition because paper replacement through e-labeling presupposes that users can access product information through digital channels [21]. It was constructed as an equally weighted composite of calibrated information and communication technology infrastructure readiness, personal digital-device adoption, and internet penetration. The remaining conditions were derived from the thematic and content analyses. The outcome, digital infrastructure readiness, policy scope, and policy maturity were treated as fuzzy sets, whereas MAH and NCA responsibilities were treated as crisp sets according to whether explicit responsibility was identified in the reviewed policy or regulatory documents.

Calibration of set membership was guided by theory, substantive case knowledge, and external benchmarks. In fuzzy-set terms, a membership score of 0.50 represents the qualitative crossover point at which a case is neither more in nor more out of the target set. Calibration therefore began by identifying the substantive transition separating categories that were more out of each set from those that were more in. For policy maturity, this boundary was actual policy implementation; for policy scope, it was coverage of at least 1 major market segment; and for the outcome, it was the possibility of omitting paper-based information under at least some circumstances. The distances between successive membership scores reflected the substantive importance of the corresponding regulatory transitions rather than assumed equal intervals. No observed category was assigned exactly 0.50. Because digital infrastructure readiness was measured on a continuous scale, it was calibrated using the direct method. For each component indicator, prespecified raw-score anchors were set at 50 for full nonmembership, 75 for the crossover point, and 99 for full membership. The crossover point of 75 was anchored to the International Telecommunication Union’s (ITU) reported global estimate of internet use in 2025 [22]. All calibration rules were specified before truth-table construction and were not adjusted in response to solution consistency or coverage. To assess the robustness of the calibration, a sensitivity analysis was conducted for the ordinal policy conditions and the outcome using 2 alternative specifications. In the crossover-compressed specification, intermediate membership scores were shifted by approximately 0.05 toward the 0.50 crossover point. Where this adjustment would have produced a score of exactly 0.50, a value of 0.49 or 0.51 was assigned to preserve the theoretically defined distinction between categories that were more out than in and those that were more in than out of the relevant set. In the contrast-enhanced specification, intermediate membership scores were shifted by approximately 0.05 away from the crossover point. The 0.05 and 0.95 anchor scores were retained in both alternative specifications.

Necessity analysis was first performed to examine whether any single condition was necessary for the outcome. Sufficiency analysis was then conducted by constructing a truth table with paper replacement as the outcome and the 5 selected conditions. The frequency threshold was set at 2 cases per configuration, and the inclusion threshold for assigning a configuration to the outcome was set at 0.80. Boolean minimization was performed using the Quine-McCluskey algorithm. Because no directional expectations were specified, the main solution was derived using conservative minimization, in which logical remainders were not included. Solutions were evaluated using standard fsQCA metrics, including consistency, proportional reduction in inconsistency (PRI), and raw coverage. Sensitivity was evaluated by comparing the resulting solution terms and their consistency, PRI, and coverage. All analyses were conducted in R software (R Foundation for Statistical Computing) using the QCA package.

### Ethical Considerations

This study used only publicly available documents and did not involve human participants, personal data, or identifiable individual-level information. In accordance with the Seoul National University Institutional Review Board Regulations, the study was not considered human subjects research and therefore did not require ethics review or approval [23]. As no participants were involved, informed consent or a consent waiver, privacy and confidentiality protections, and participant compensation were not applicable. The manuscript and supplementary materials contain no images or information that could identify individual participants or users. All data sources used in the study are provided in Tables S1 and S2 in Multimedia Appendix 1.

## Results

### Eligible Policy Documents

**Figure 1:**
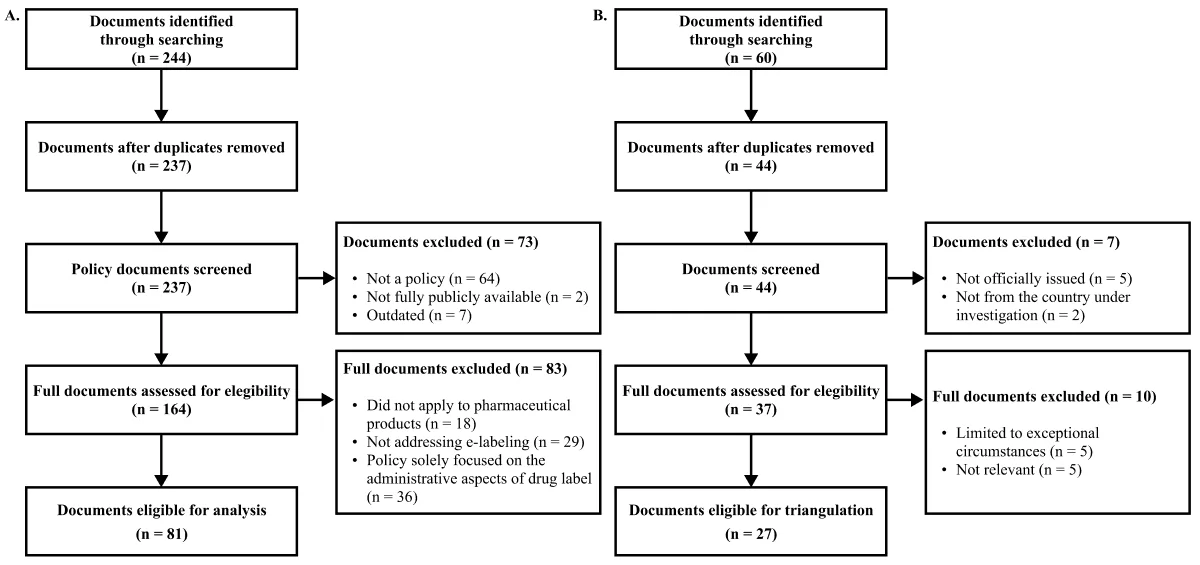
PRISMA-S (Preferred Reporting Items for Systematic Reviews and Meta-Analyses literature search extension)–adapted flowchart of the document selection process. (A) Main corpus: selection of pharmaceutical e-labeling policy documents selection from International Coalition of Medicines Regulatory Authorities member jurisdictions. (B) Triangulation corpus: selection of e-labeling-relevant documents from international stakeholder organizations.

In the early phase of the investigation, we identified 164 policy documents from all ICMRA member jurisdictions. After full-text assessment, we excluded 18 documents that did not apply to pharmaceutical products, 29 that did not address e-labeling, and 36 that focused solely on the administrative aspects of drug labeling. The remaining 81 policy documents from 20 jurisdictions were included in the main analysis (Figure 1A). No eligible publicly accessible official documents were identified for India or Nigeria under the predefined inclusion criteria; therefore, these jurisdictions were excluded from the main comparative analysis. Among these documents, 8 were classified as having legal authority, while the remaining 73 were categorized as nonlegal guidance documents. For triangulation, 37 relevant stakeholder documents were identified. Of these, 5 documents addressing e-labeling under exceptional circumstances and 5 documents considered less directly relevant were excluded. Finally, 27 documents were included in the triangulation corpus (Figure 1B).

### Identified Themes and Coding Framework

**Figure 2:**
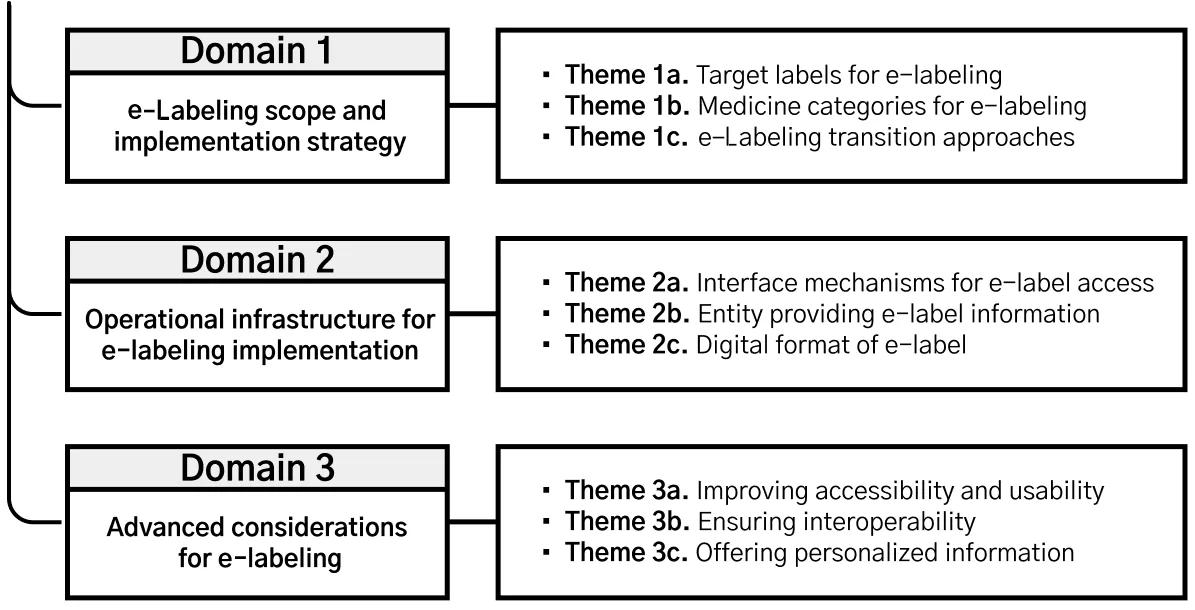
Qualitative coding framework for e-labeling policy analysis. The figure presents the coding tree used for the thematic and qualitative analysis of e-labeling policies. The coding framework consisted of three predefined domains (deductive), within which specific themes were inductively derived from the data. This hybrid framework was subsequently applied to systematically analyze and categorize policy documents.

Any discrepancies in the coding process of the hybrid thematic analysis were resolved through discussion, and mutual agreement was reached on all divergent parts. As a result, the final coding achieved a Cohen κ value of at least 0.95, indicating a high level of intercoder reliability. The coders inductively derived 9 core themes from the main policy corpus and subsequently organized them deductively into 3 predefined analytic domains for e-labeling implementation (Figure 2). The first domain addressing the scope and implementation strategies of e-labeling yielded three themes: (1a) target labels, (1b) medicine categories, and (1c) transition approaches. The second domain, dealing with the operational infrastructure required for e-labeling implementation, included three themes: (2a) interface mechanisms, (2b) entity providing information, and (2c) presenting digital formats. Finally, the third domain, covering further considerations, included the themes (3a) accessibility and usability, (3b) interoperability, and (3c) personalization.

In the main policy corpus, the most frequently coded themes were concentrated in domain 1 and the basic operational elements of domain 2 (Table S3 in Multimedia Appendix 1). Medicine categories (theme 1b) were coded in 66/81 (81.5%) documents, followed by target labels for e-labeling (theme 1a; 64/81, 79.0%), interface mechanisms (theme 2a; 59/81, 72.8%), transition approaches (theme 1c; 54/81, 66.7%), and entity providing e-labels (theme 2b; 52/81, 64.2%). By contrast, digital format (theme 2c) was coded in fewer than one-third of main policy documents (24/81, 29.6%). Within domain 3, accessibility and usability (theme 3a) were more frequently addressed (48/81, 59.3%) than interoperability (theme 3b; 14/81, 17.3%) and personalized information (theme 3c; 10/81, 12.3%).

The triangulation corpus confirmed the relevance of all 9 core themes, while showing greater emphasis on transition and system-level issues. Transition approaches (theme 1c) were coded in 24/27 (88.9%) triangulation documents, followed by medicine categories (theme 1b) and entity providing e-labels (theme 2b), each coded in 20/27 (74.1%) documents. Compared with the main policy corpus, triangulation documents more frequently addressed interoperability (theme 3b; 14/27, 51.9%) and personalized information (theme 3c; 7/27, 25.9%). Along with the frequent coding of transition approaches, medicine categories, and entity providing e-labels, this pattern highlighted the importance of policy scope, responsibility structure, and advanced functions in the transition toward potential paper replacement. Cross-cutting codes were retained outside domains 1-3 because they were identified only in the triangulation corpus. These codes were regulatory life cycle efficiency and timely updates (4/27, 14.8%) and implementation risks and transition safeguards (5/27, 18.5%). They were treated as contextual triangulation findings that highlighted broader stakeholder concerns about update efficiency, regulatory process optimization, and transition safeguards.

### Content Analysis: Cross-National Comparison of e-Labeling Policy

#### Domain 1: e-Labeling Scope and Implementation Strategy

Domain 1 examined how jurisdictions framed pharmaceutical e-labeling in terms of target product information, covered medicine categories, and transition approaches from paper-based to electronic formats (Table 1). The target information for theme 1a was different depending on the jurisdiction specific regulatory terminology. Most policies addressed PIs, package leaflets, patient information leaflets or other product-information documents. Some jurisdictions, including Canada and the European Union (EU), covered broader statutory product-information components, such as labeling and product monographs and summaries of product characteristics. Medicine coverage for theme 1b varied from narrow product groups to wide medicine categories. Some jurisdictions took targeted approaches such as authority-designated prescription medicines (Republic of Korea), health care professional (HCP)–administered injectables (Australia and Switzerland), or selected medicines (France and Brazil). Other categories were more general, such as prescription and nonprescription medicines or all medicines authorized in the jurisdiction. The policy tendency was generally toward a phased or targeted expansion, not an instant application to all medicines. The greatest variation in the correlation between the electronic and paper-based information was regarding theme 1c, transition approaches. In many jurisdictions, electronic information was used in addition to printed product information and paper leaflets or statutory information were still required. Others were in the form of parallel or dual format (eg, China has provided the electronic PIs and maintained full or simplified paper inserts). In the case of a smaller group, paper information could be substituted conditionally or limited, for example, the Government of Brazil’s pilot-based digital substitution with a print-on-demand guarantee, the Republic of Korea’s substitution for Ministry of Food and Drug Safety–designated prescription medicines, Switzerland’s omission of paper information for certain products administered by HCPs, and Japan’s electronic-default approach for prescription or medical-use medicines.

Overall, for domain 1, there were large differences between countries in the definition, scope and positioning of e-labeling compared to paper-based information. The main regulatory gaps included inconsistent terminology, uneven coverage of medicines and limited movement beyond supplementary or dual-format approaches in many jurisdictions.

**Table 1:** Description of national e-labeling policies addressing scope and implementation strategy (domain 1).

Country/region	Target labels for e-labeling (theme 1a)	Medicine categories (theme 1b)	Transition approaches (theme 1c)	Policy type
Brazil	Digital package leaflet	Eligible medicines marketed for ≥5 years; free samples; medicines for health establishments; government/SUS^a^ medicines; nonprescription medicines in multiple packaging	Pilot-based digital substitution with print-on-request guarantee	Legal for themes 1a/1b/1c
Canada	Drug labels; PIs^b^; inner/outer labels; product monographs; dispensing materials; Canadian Drug Facts Table	Human prescription and nonprescription drugs	Supplementary electronic links on paper labels; mandatory key information on outer labels	Nonlegal for themes 1a/1b/1c
Mexico	Patient instruction leaflet	OTC^c^	Optional QR-code access	Proposed rule for themes 1a/1c
United States	Patient Medication Information	Outpatient human prescription drugs	Patient-choice electronic PMI^d^; paper default	Proposed regulation for themes 1a/1b/1c
Republic of South Africa	PI for patients, consumers, and HCPs^e^	OTC; Schedule 0-2 products; planned prescription-only expansion	Supplementary SAHPRA^f^ online access	Nonlegal for themes 1a/1b/1c
China	Drug package inserts	Selected oral, external-use, and other preparations	Parallel electronic inserts; retained full/simplified paper inserts	Legal for themes 1a/1b/1c
Japan	Package inserts	Prescription/medical-use medicines; OTC and direct consumer-purchased products excluded	Electronic-default provision; paper retained for OTC/direct consumer-purchased products	Legal for themes 1a/1b/1c
Republic of Korea	Package insert	MFDS^g^-specified prescription medicines; hospital-administered injectables; biologics	Digital substitution for MFDS-designated prescription medicines	Legal for themes 1a/1b/1c
Singapore	HSA^h^-approved electronic PI and/or PIL^i^	POM^j^ therapeutic products; draft extension to Pharmacy-only and GSL^k^ therapeutic products	ePI^l^/PIL distribution with or without physical printed copies	Nonlegal for themes 1a/1b/1c
European Union	Statutory product information; SmPC^m^; Annex II; labeling; PL^n^ priority	All human medicines in EU^o^/EEA^p^; OTC and prescription-only products	Nonreplacement of statutory information; PL/packaging information retained	Nonlegal for themes 1a/1b/1c
European Union	SmPC; labeling; PL	All Union-authorized human medicines; CAPs^q^ and NAPs^r^	Member-state paper/electronic/both PL; free print copy for electronic-only provision	Proposed directive for themes 1a/1b/1c
United Kingdom	Patient information leaflets	Medicinal products	N/A^s^	Proposed project for themes 1a/1b; nonlegal
France	PL, SmPC	Selected nonhospital medicines; hospital-sector medicines with exclusions.	Complementary e-leaflet; hospital paper-removal test; community removal discussion	Nonlegal for themes 1a/1b/1c
Germany	PIL, SmPC	Approved medicinal products	N/A	Nonlegal for themes 1a/1b
Germany	Statutory information—the approved PI; information in the labeling and/or the PL	Both OTC and prescription only products	Nonreplacement of statutory information/printed PL	Nonlegal for themes 1a/1b/1c; following EU [24]
Italy	SmPCs, PL, and Product Information Sheets	All medicines authorized in Italy	Paper PIM^t^ inside packaging	Nonlegal for themes 1a/1b/1c
The Netherlands	Mandatory product information: PIL, SmPC, and labeling	All authorized/to-be-authorized medicinal products	Dutch PL always inserted in packaging	Legal for themes 1a/1b/1c
The Netherlands	Statutory information: the approved PI; information in the labeling and/or the PL	Both OTC and prescription only products	Nonreplacement of statutory information/printed PL	Nonlegal for themes 1a/1b/1c; following EU [24]
Ireland	Statutory information: approved PL, SmPC, PI, labeling	CAPs; both OTC and prescription only products	Nonreplacement of statutory information	Nonlegal for themes 1a/1b/1c; following EU [24,25]
Sweden	Product information—SmPC, PL, labeling	Medicines	ePI not replacing paper leaflet	Nonlegal for themes 1a/1b/1c
Switzerland	Information for HCPs	HCP-administered medicinal products; injectables; infusions	PL/PI omission; paper or barcode-linked electronic provision	Legal for themes 1a/1b/1c
Australia	PI for HCP-administered injectable medicines; CMI^u^ excluded	HCP-administered injectables; self/carer-administered injectables excluded	Supplementary electronic medicine information only	Legal for themes 1a/1b/1c
Australia	Preparation instructions for injectable medicines	HCP-administered injectable medicines	Proposed electronic substitution of printed preparation instructions; accessibility considerations	Proposed rule for themes 1a/1b/1c
New Zealand	CMI	Medicines supplied in New Zealand	Supplementary QR/website access to approved medicine information	Nonlegal for themes 1a/1c

^a^SUS: Sistema Único de Saúde (Brazilian public health system).

^b^PI: package insert/product information.

^c^OTC: over the counter products.

^d^PMI: patient medication information.

^e^HCP: health care professional.

^f^SAHPRA: South African Health Products Regulatory Authority.

^g^MFDS: Ministry of Food and Drug Safety.

^h^HSA: Health Sciences Authority.

^i^PIL: patient information leaflet.

^j^POM: prescription-only medicines.

^k^GSL: general sale list.

^l^ePI: electronic product information.

^m^SmPC: summary of product characteristics.

^n^PL: package leaflet.

^o^EU: European Union.

^p^EEA: European Economic Area.

^q^CAP: centrally authorized products.

^r^NAP: nationally authorized products.

^s^N/A: not applicable.

^t^PIM: Product Information Sheet.

^u^CMI: Consumer Medicine Information.

#### Domain 2: Operational Infrastructure for e-Labeling Implementation

Domain 2 focused on the operational infrastructure for e-labeling implementation, including the interface mechanisms, the entities responsible for the delivery of e-labels, and the digital formats (Table 2). In most jurisdictions for theme 2a, practical access mechanisms were specified to link physical packaging to electronic information. QR codes, 2-dimensional barcodes, data matrix codes, URLs, short links or other machine-readable codes were commonly used. Some policies specified technical requirements more precisely, such as Japan’s use of Global Standards 1 (GS1)–based code formats and Global Trade Item Number (GTIN) product codes, while others made more general remarks regarding QR codes or similar machine-readable mechanisms. However, some policies did not clearly specify interface mechanisms, leading to uneven operational detail across jurisdictions. For theme 2b, there were different policies regarding who was responsible for providing, hosting or maintaining e-label information. In a number of jurisdictions, the responsibility was placed mainly on MAHs, as reflected in policies from Brazil, Canada, China, the Republic of Korea, Singapore, Switzerland, Australia, and New Zealand. Other jurisdictions used regulatory authority websites, public databases or official information systems such as Japan’s Pharmaceuticals and Medical Devices Agency (PMDA) Safety Information Posting System, France’s public medicines database under the French National Agency for the Safety of Medicines and Health Products (ANSM) authority, Germany’s PharmNet.Bund, and Italian Medicines Agency’s (AIFA) website or medicines database app. The EU adopted a more platform-based model. In theme 2c, digital format was not specified as consistently as access mechanisms or responsible entities. Some jurisdictions permitted or specified document-based formats such as noneditable PDF or HTML in Brazil and editable PDF in Canada. Others spoke about more structured or machine-readable formats such as Health Level 7 (HL7) Structured Product Labeling (SPL) in the United States, machine-readable formats in Singapore, and Fast Healthcare Interoperability Resources (FHIR)–based or common electronic standards in the EU, Sweden, and the Netherlands. By contrast, some policies offered no specification of a digital format, even when they described interface mechanisms or responsible entities.

In general, domain 2 showed that operational infrastructure for e-labeling was most developed in relation to access mechanisms and responsibility allocation, while digital format and technical standardization remained less consistently addressed. The main regulatory gaps were unclear interface specifications in some jurisdictions, variation in whether e-labels were managed by industry actors, authorities, or shared platforms, and limited specification of structured digital formats.

**Table 2:** Description of national e-labeling policies addressing operational infrastructure (domain 2).

Country/region	Interface mechanisms (theme 2a)	Entity providing e-label (theme 2b)	Digital format (theme 2c)	Policy type
Brazil	QR/digital mechanism on primary packaging for multiple packaging	Registration-holder-managed RIEP^a^; Anvisa-authorized links; approved information; storage/versioning/availability/traceability/compliance responsibility	Noneditable PDF or HTML	Legal for themes 2a/2b/2c
Mexico	Optional QR code	N/A^b^	N/A	Proposed regulation for theme 2a
United States	N/A	FDA^c^ public labeling repository; manufacturer-submitted prescribing information; manufacturer/government websites	SPL^d^; XML-based HL7^e^	Proposed rule for themes 2b/2c
Canada	QR code, 2D matrix code, website link, or URL-based access	Sponsor/manufacturer/license-holder platforms; approved information; URL redirection and label QA responsibility	Editable PDF from electronic sources	Nonlegal for themes 2a/2b/2c
Republic of South Africa	Matrix barcode/QR code; URL; contact details	SAHPRA^f^ Online Medicines Directory; applicant/MAHs^g^/HCR^h^ updating responsibility	N/A	Nonlegal for themes 2a/2b
China	Barcode/QR code on smallest sales-unit packaging or paper insert	MAHs implementation and maintenance; NMPA^i^/provincial oversight	Simple image conversion (eg, JPG) not recommended	Legal for themes 2a/2b/2c
Japan	Outer-packaging codes with GTIN^j^; GS1^k^-based code formats	MAHs registration/publication; PMDA Safety Information Posting System; PMDA website	N/A	Legal for themes 2a/2b
Republic of Korea	QR code; barcode; other container/package codes	MAHs-operated e-label website; company website/equivalent platform linkage	N/A	Legal for theme 2a; nonlegal for themes 2b
Singapore	URL; short link; QR code; machine-readable code	Registrant-managed hosting platform; HSA^l^-approved PI^m^/PIL^n^; availability/update alignment responsibility	Machine-readable digital format	Nonlegal for themes 2a/2b/2c
European Union	2D barcode/QR code/NFC^o^; URL provision	PLM^p^ portal; EMWP^q^; NAP^r^ national websites; MAHs creation/editing/submission; EMA^s^/NCA^t^ approval/publication	HTML; EU^u^ ePI^v^ common standard; FHIR^w^	Nonlegal for themes 2a/2b/2c
European Union	Digitally readable outer-packaging barcode	Agency/member-state/Commission EMWP setup and maintenance	Common electronic standards for PL, SmPC^x^, and labeling	Proposed directive for themes 2a/2b/2c
United Kingdom	N/A	N/A	N/A	N/A
France	QR codes on medicine boxes	ANSM^y^ authority; public medicines database (BDPM^z^)	N/A	Nonlegal for themes 2a/2b
Germany	QR codes; linear barcode; 2D Data Matrix; NFC	PharmNet.Bund medicinal product information system	N/A	Nonlegal for themes 2a/2b
Germany	2D barcode/QR code/NFC; URL provision	MAHs/NCA/third-party maintenance; NCA websites; statutory/complementary platforms; stand-alone PDF	Website, platform, PDF	Nonlegal for themes 2a/2b/2c; following EU [24]
Italy	2D barcode, or QR code with URL	AIFA^aa^ website or drug database app “Medicinali”	N/A	Nonlegal for themes 2a/2b
The Netherlands	QR-equivalent techniques; 2D barcode; Data Matrix; full URL	MAHs-provided QR-linked information	Can be determined freely.	Legal for themes 2a/2b/2c
The Netherlands	N/A	ePI Authoring portal on PLM portal	Common EU electronic standard, FHIR	Nonlegal for themes 2a/2b/2c; as pilot project with EU
The Netherlands	2D barcode/QR code/NFC; URL provision	MAHs/NCA/third-party maintenance; NCA websites; statutory/complementary platforms; stand-alone PDF	Website, platform, PDF	Nonlegal for themes 2a/2b/2c; Following EU [24]
Ireland	QR code or 2D barcode	N/A	N/A	Nonlegal for theme 2a
Ireland	2D barcode/QR code/NFC; URL provision	MAHs availability/update responsibility; MAHs/NCA/third-party maintenance; NCA websites/platforms/stand-alone PDF	Website, platform, PDF	Nonlegal for themes 2a/2b/2c; Following EU [24,25]
Sweden	N/A	Swedish MPA website; PLM portal	FHIR adapted structured format (fixed headings, controlled vocabularies)	Nonlegal for themes 2b/2c
Switzerland	Packaging barcode/QR code with URL	MAHs integrity and accessibility responsibility	N/A	Legal for themes 2a/2b
Australia	QR/machine-readable codes on labels; supplementary access	TGA^ab^ website for approved PI; sponsor-provided supplementary information	N/A	Legal for themes 2a/2b
Australia	QR-linked approved information; GTIN-based machine-readable codes	Sponsor-controlled unrestricted QR-linked website; TGA-approved content consistency	N/A	Proposed rule for themes 2a/2b
New Zealand	QR code; website address; electronically readable code; GS1 barcode/GTIN reference	Medsafe or company website; sponsor consistency responsibility	N/A	Nonlegal for themes 2a/2b

^a^RIEP: Repositório Institucional de Estudos e Pesquisas Educacionais.

^b^N/A: not applicable.

^c^FDA: Food and Drug Administration.

^d^SPL: Structured Product Labeling.

^e^HL7: Health Level 7.

^f^SAHPRA: South African Health Products Regulatory Authority.

^g^MAH: marketing authorization holder.

^h^HCR: holder of certificate of registration.

^i^NMPA: National Medical Products Administration.

^j^GTIN: Global Trade Item Number.

^k^GS1: Global Standards 1.

^l^HSA: Health Sciences Authority.

^m^PI: package insert/product information.

^n^PIL: patient information leaflet.

^o^NFC: near field communication.

^p^PLM: EU product life cycle management.

^q^EMWP: European Medicines Web Portal.

^r^NAP: nationally authorized product.

^s^EMA: European Medicines Agency.

^t^NCA: national competent authority.

^u^EU: European Union.

^v^ePI: Electronic product information.

^w^FHIR: Fast Healthcare Interoperability Resources.

^x^SmPC: summary of product characteristics.

^y^ANSM: French National Agency for the Safety of Medicines and Health Products.

^z^BDPM: Base de Données Publique des Médicaments (Public Database of Medicines).

^aa^AIFA: Agenzia Italiana del Farmaco (the Italian Medicines Agency).

^ab^TGA: Therapeutic Goods Administration.

#### Domain 3: Advanced Considerations for e-Labeling

Domain 3 compared whether e-labeling policies addressed advanced considerations beyond basic electronic access, including accessibility and usability, interoperability, and personalized information (Table 3). For theme 3a, accessibility and usability were the most common advanced considerations. Most policy content focused on making electronic information easier to access, read, and use. These measures often focused on patients and users with access needs. These included clearer access routes, support for readability, multimedia materials, multilingual access and disability-oriented formats. Brazil had one of the most comprehensive approaches with assistive content, continuous access, contingency planning and measures aimed at disability. Fewer jurisdictions addressed theme 3b, interoperability. It was mostly described as the use of structured data standards or as linkage to larger digital health systems. Standards-based or system linked approaches were taken by the US, the EU, the Netherlands, Sweden and Brazil on interoperability. These included HL7 SPL, FHIR, the EU ePI common standard, controlled vocabularies and linkage to health data platforms. In the majority of the remaining jurisdictions, interoperability-related policy content was not specified. The least discussed theme for theme 3c was personalized information. It was described, where mentioned, as an advanced function used for tailoring, filtering or delivering product information based on user or patient-specific requirements. The EU, the Netherlands (as a pilot participating country), and Sweden addressed this theme through user preferences, treatment management, update alerts, or personalized information extraction. Brazil and the US referred to personalized or patient-centric applications mainly as potential functions of future system integration.

Overall, domain 3 showed that advanced policy considerations were unevenly developed. Accessibility and usability were the most common areas of policy attention, whereas interoperability and personalized information were addressed in a smaller group of jurisdictions. This imbalance indicates a regulatory gap between improving user access to electronic information and developing e-labeling as an interoperable, personalized component of digital health systems.

**Table 3:** Description of national e-labeling policies addressing advanced considerations (domain 3).

Country/region	Accessibility and usability (theme 3a)	Interoperability (theme 3b)	Personalized information (theme 3c)	Policy type
Brazil	Readability/comprehension design; audio/video/assistive content; open/free 24/7 RIEP^a^ access; contingency planning; QR instructions; disability-access measures	RIEP interoperability; RNDS^b^ linkage; technical standards; controlled vocabularies; FHIR^c^; IDMP^d^; medicine ontology	Adverse-event reporting link; no user identification/personal data/promotional use; personalized information potential via electronic product information/future integration	Legal for themes 3a/3b/3c
Mexico	N/A^e^	N/A	N/A	N/A
United States	N/A	HL7^f^ SPL^g^/CDA^h^; searchable, transferable, integrable labeling; emerging FHIR exchange	FHIR-enabled EHR^i^/wearable/dataset integration; personalized/patient-centric applications	Nonlegal for themes 3b/3c
Canada	Multiple access methods; clear instructions; plain-language/readable design; WCAG^j^-aligned accessibility; text alternatives; search; multilingual usability	N/A	N/A	Nonlegal for theme 3a
Republic of South Africa	Visually impaired access; enlarged font; audio-perceptible formats	N/A	N/A	Nonlegal for theme 3a
China	Age-friendly/barrier-free inserts; readability improvement; zoom/consistent display; large fonts/icons; high contrast; optional audio/Braille	N/A	N/A	Legal for theme 3a
Japan	User-support materials; easy-to-understand leaflets; explanatory videos	N/A	N/A	Nonlegal for theme 3a
Republic of Korea	Vulnerable-user accessibility; communication-failure alternatives; version/applicability notice; previous-version access	N/A	N/A	Nonlegal for theme 3a
Singapore	Dedicated link access; minimized navigation; online instructional videos	N/A	N/A	Nonlegal for theme 3a
European Union	Multilingual access; versioning; expiry/update alerts; searchability; videos; large fonts; accessibility standards; high contrast; audio/screen readers	FHIR; EU^k^ ePI^l^ common standard; electronic health care/EHR/e-prescribing integration	Personalized information; search retrieval; user preferences; treatment management; update alerts	Nonlegal for theme 3a/3b/3c
European Union	FHIR XML translation at PLM^m^ portal	Full NCA^n^ interoperability support	N/A	Proposed nonlegal for theme 3a/3b
United Kingdom	N/A	N/A	N/A	N/A
France	Additional documents/videos; risk-minimization materials; voice-over	N/A	N/A	Nonlegal for theme 3a
Germany	Additional information (eg, video); useful/nonpromotional content	N/A	N/A	Nonlegal for theme 3a; following EU [24]
Italy	N/A	N/A	N/A	N/A
The Netherlands	Pharmacovigilance-related information; educational material	N/A	N/A	Legal for theme 3a
The Netherlands	Automatic update notifications; supplementary video/audio content	FHIR-based EU common standard; adverse-event reporting integration; health-system/Medication Process exchange discussions	Adaptation of product information to individual needs	Nonlegal for themes 3a/3b/3c; as pilot project with EU
The Netherlands	Additional information (eg, video); useful/nonpromotional content	N/A	N/A	Nonlegal for theme 3a; following EU [24]
Ireland	All EU official languages; additional information (eg, video)	N/A	N/A	Nonlegal for theme 3a; following EU [24,25]
Sweden	Search functionalities	FHIR compatibility; e-health system use/combination	Personalized extraction; automated comparison across medicines (eg, interactions, excipients)	Nonlegal for themes 3a/3b/3c
Switzerland	Three official languages; minimum 7-point font; medicine-use information/videos	N/A	N/A	Legal for theme 3a
Australia	Link-destination indication; short URLs; instructional videos; cross-platform/device access; faster access/readability	N/A	N/A	Legal for theme 3a
Australia	QR-purpose statement; unrestricted access; no login/payment/geoblocking/barriers	N/A	N/A	Proposed rule for theme 3a
New Zealand	QR-enabled consumer/patient access; purpose statement near QR code	N/A	N/A	Nonlegal for theme 3a

^a^RIEP: Repositório Institucional de Estudos e Pesquisas Educacionais.

^b^RNDS: Rede Nacional de Dados em Saúde (National Health Data Network).

^c^FHIR: Fast Healthcare Interoperability Resources.

^d^IDMP: Identification of Medicinal Products.

^e^N/A: not applicable.

^f^HL7: Health Level 7.

^g^SPL: Structured Product Labeling.

^h^CDA: Clinical Document Architecture.

^i^EHR: electronic health record.

^j^WCAG: Web Content Accessibility Guidelines.

^k^EU: European Union.

^l^ePI: electronic product information.

^m^PLM: EU product life cycle management.

^n^NCA: national competent authority.

### e-Labeling Policy Maturity Level Across Jurisdictions

The maturity assessment indicated considerable variation in the development of e-labeling regulation across jurisdictions (Table 4). Classification was based on the predefined maturity framework, which considered implementation status, regulatory authority, and policy expansion. Brazil and the Netherlands were classified as level 4, which indicates legally based implementation with broader expansion or more advanced considerations. The classification of the Netherlands partly reflected its alignment with EU-level regulatory requirements and advanced ePI initiatives explicitly referenced in the policy materials. China, Japan, the Republic of Korea, Australia, and Switzerland were classified as level 3, reflecting implemented e-labeling policies supported by a legal basis. Canada, South Africa, Singapore, France, Germany, Italy, Sweden, and New Zealand were classified as level 2, indicating implementation mainly supported by nonlegal policy instruments. Mexico, the United States, the United Kingdom, and Ireland were classified as level 1, reflecting planning, proposal, or limited implementation status. The EU was presented descriptively as a regional regulatory framework and was not assigned a jurisdiction-level maturity score.

**Table 4:** Jurisdiction-level basis for e-labeling regulatory maturity assignment.

Country/region	Implementation status	Level of authority	Expansion	Maturity level^a^
Brazil	Implemented	Legal	Yes	Level 4
Mexico	Planning/proposed	Proposed	No	Level 1
United States	Planning/proposed	Proposed	Yes	Level 1
Canada	Implemented	Nonlegal	No	Level 2
Republic of South Africa	Implemented	Nonlegal	No	Level 2
China	Implemented	Legal	No	Level 3
Japan	Implemented	Legal	No	Level 3
Republic of Korea	Implemented	Legal	No	Level 3
Singapore	Implemented	Nonlegal	No	Level 2
European Union	Implemented	Legal (proposed)	Yes	—^b^
United Kingdom	Planning/proposed	Nonlegal	No	Level 1
France	Implemented	Nonlegal	No	Level 2
Germany	Implemented	Nonlegal	No	Level 2
Italy	Implemented	Nonlegal	No	Level 2
Netherlands	Implemented	Legal	Yes	Level 4
Ireland	Not implemented	Nonlegal	No	Level 1
Sweden	Implemented	Nonlegal	Yes	Level 2
Switzerland	Implemented	Legal	No	Level 3
Australia	Implemented	Legal	No	Level 3
New Zealand	Implemented	Nonlegal	No	Level 2

^a^Maturity levels were assigned through qualitative interpretation of implementation status, level of authority, and expansion, rather than by a simple additive score. Level 1 indicates fragmented or preimplementation status; level 2 indicates implementation mainly supported by nonlegal instruments; level 3 indicates implementation with a legal basis; and level 4 indicates legally grounded implementation with broader expansion or advanced implementation considerations. Expansion was defined as the presence of policy content addressing advanced considerations under domain 3, specifically interoperability and the offering of personalized information, corresponding to themes 3b and 3c, respectively.

^b^Not available.

### fsQCA of Paper-Label Replacement

#### Distribution of Calibrated Conditions

The final fsQCA dataset consisted of 19 jurisdictions. Nigeria and India were excluded because no eligible e-labeling policy documents were identified. The EU was not treated as an independent fsQCA case, but EU-level policy content was considered when EU member-state documents explicitly referred to binding EU rules or guidance. The calibrated set-membership scores for the outcome and the 5 conditions are shown in Table 5, and the raw variables and the source references are given in Table S4 in Multimedia Appendix 1. For the outcome set, paper-label replacement, 7 jurisdictions were above the crossover point, including 6 jurisdictions with scores of 0.85 or 0.95. Twelve other jurisdictions scored below or at the crossover level. The digital infrastructure readiness ranged from 0.69 to 0.94, and all the jurisdictions were above the crossover point. The maturity of e-labeling policy ranged from 0.25 to 0.95 and the scope of e-labeling policy from 0.20 to 0.95. The crisp-set conditions also varied across jurisdictions. Explicit MAH responsibility was present in 12 jurisdictions and absent in 7 jurisdictions. Explicit NCA responsibility was present in 10 jurisdictions and absent in 9. This distribution indicated that digital infrastructure readiness was consistently high across the study jurisdictions, while the other conditions provided more cross-jurisdictional variation for the fsQCA model.

**Table 5:** Calibrated condition scores included in fuzzy-set qualitative comparative analysis model for paper-label replacement.

Country/region	Policy maturity^a^	e-Labeling policy scope^b^	MAH^c^ responsibility^d^	NCA^e^ responsibility^d^	Digital infrastructure readiness^f^	Paper-label replacement^g^
Brazil	0.95	0.40	1	0	0.76	0.85
Mexico	0.25	0.60	0	0	0.69	0.25
United States	0.25	0.60	1	1	0.93	0.25
Canada	0.55	0.95	1	0	0.89	0.25
Republic of South Africa	0.55	0.80	0	1	0.72	0.25
China	0.85	0.40	1	0	0.86	0.45
Japan	0.85	0.60	0	1	0.87	0.95
Republic of Korea	0.85	0.20	1	0	0.93	0.85
Singapore	0.55	0.95	1	0	0.94	0.65
United Kingdom	0.25	0.80	0	0	0.92	0.05
France	0.55	0.60	0	1	0.88	0.85
Germany	0.55	0.95	1	1	0.87	0.25
Italy	0.55	0.95	0	1	0.87	0.25
Netherlands	0.95	0.95	1	1	0.91	0.25
Ireland	0.25	0.80	1	1	0.92	0.25
Sweden	0.55	0.95	0	1	0.92	0.25
Switzerland	0.85	0.20	1	0	0.93	0.85
Australia	0.85	0.20	1	0	0.93	0.85
New Zealand	0.55	0.80	1	1	0.90	0.25

^a^Policy maturity: level 1=0.25, level 2=0.55, level 3=0.85, and level 4=0.95.

^b^0.20=narrow/HCP-specific or designated subset; 0.40=selected medicines/eligible groups; 0.60=1 major market segment; 0.80=near-all medicines with information-type or institutional restrictions; 0.95=all/near-all medicines with major statutory product-information components.

^c^MAH: marketing authorization holder.

^d^Crisp sets: 1=explicit responsibility identified; 0=no explicit responsibility identified.

^e^NCA: national competent authority.

^f^Mean of calibrated information and communications technology infrastructure readiness, personal digital-device adoption, and internet penetration. For the calibration of each component indicator, anchors were set at 50 for full nonmembership, 75 for the crossover point, and 99 for full membership.

^g^Outcome (paper-label replacement): 0.05=no evidence; 0.25=supplementary/nonsubstitutive; 0.45=structured parallel provision; 0.65=flexible dual-format with possible paper omission; 0.85=conditional/limited substitution; 0.95=electronic-first/default substitution.

#### Configurational Conditions for Paper-Label Replacement

Necessity analysis is provided in Table S5 in Multimedia Appendix 1. The highest values of necessity consistency were obtained for digital infrastructure readiness and e-labeling policy maturity with consistency scores of 0.980 and 0.944, respectively. Both values were over the conventional threshold of 0.90 for necessity consistency. However, the coverage of digital infrastructure readiness and relevance of necessity was lower than that of e-labeling policy maturity. This pattern reflected the limited variation in digital infrastructure readiness, with all jurisdictions having calibrated scores above the crossover point. e-Labeling policy maturity showed high necessity consistency together with comparatively higher coverage and relevance. All other conditions and their negations had lower necessity consistency values. The retained truth table is presented in Table S6 in Multimedia Appendix 1. Six observed configurations were above the threshold frequency of 2 cases per configuration. Two configurations were assigned to the outcome set when the inclusion threshold was set to 0.80. The first outcome-assigned configuration included Australia, Switzerland, the Republic of Korea, Brazil, and China and the second was Canada and Singapore. Conservative minimization yielded 1 sufficient configuration for paper-label replacement. This configuration consisted of the presence of digital infrastructure readiness, e-labeling policy maturity, and explicit MAH responsibility, combined with the absence of explicit NCA responsibility. Solution consistency was 0.867, PRI was 0.806, and raw coverage was 0.515 (Table 6). The same sufficient configuration was retained under both the crossover-compressed and contrast-enhanced calibration specifications. Across the sensitivity analyses, consistency ranged from 0.844 to 0.896, PRI from 0.796 to 0.826, and raw coverage from 0.491 to 0.542, supporting the robustness of the identified configuration to alternative membership-score assignments. The consistency values indicated that the retained configuration was consistently associated with membership in the paper-label replacement outcome set, while the raw coverage values indicated that it accounted for approximately half of the outcome-set membership.

**Table 6:** Fuzzy-set qualitative comparative analysis findings for paper-label replacement under baseline and alternative calibration specifications^a^.

Configuration	Main solution (baseline)	Crossover-compressed	Contrast-enhanced
Contextual condition			
Digital infrastructure readiness	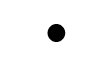 ^b^	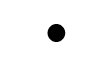	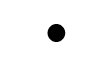
Policy-intrinsic conditions			
e-Labeling policy scope	—^c^	—	—
e-Labeling policy maturity	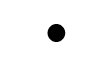	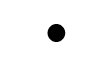	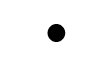
MAH^d^ responsibility	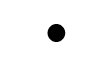	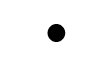	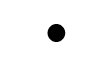
NCA^e^ responsibility	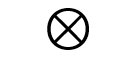 ^f^	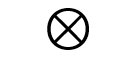	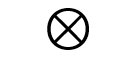
Consistency	0.867	0.896	0.844
PRI^g^	0.806	0.826	0.796
Raw coverage	0.515	0.491	0.542

^a^Because a single solution term was obtained, overall solution consistency and coverage are identical to the solution-level consistency and coverage.

^b^Indicates the presence of a condition.

^c^Indicates “don’t care” conditions.

^d^MAH: marketing authorization holder.

^e^NCA: national competent authority.

^f^Indicates the absence of a condition.

^g^PRI: proportional reduction in inconsistency.

## Discussion

### Principal Findings

This study examined the current status of pharmaceutical e-labeling policies and the conditions associated with progress toward replacing paper labels. Using policy document analysis and fsQCA, we analyzed legal and nonlegal policy documents from ICMRA member jurisdictions across 3 domains. The findings show that pharmaceutical e-labeling has advanced across many jurisdictions, but its regulatory development remains fragmented.

Among the jurisdictions included in the study, 63.2% (12/19) were classified as level 1 or level 2, and only 7 countries had developed legally binding frameworks. Brazil and the Netherlands were classified as the highest maturity level due to policies that integrated implementation, a legal basis, and expansion toward advanced functions. This framework-based maturity assessment is broadly consistent with external industry reviews and APAC-Asia materials, which have described Brazil, Japan, Australia, and the EU as relatively advanced cases of e-labeling implementation [18,26,27]. These findings suggest that, although e-labeling is increasingly recognized as more than a digitized version of paper labeling, it has not yet been fully institutionalized as a routine component of national digital health governance [1]. This is important because e-labeling can provide trusted information and function as a regulatory and health data layer within broader health information systems [2]. Therefore, strengthening legal provisions, harmonizing definitions and technical expectations, and clarifying responsibilities are critical steps toward embedding e-labeling within mature, interoperable, and resilient digital health ecosystems.

Most countries have adopted a stepwise approach to e-labeling coverage and paper replacement. This approach appears appropriate given that e-labeling affects users differently depending on the medicine category, care setting, and degree of HCP supervision. Products administered by HCPs, such as injectable medicines, and prescription drugs were often the initial candidates for e-labeling. In most jurisdictions, e-labeling was suggested as a supplementary material rather than a substitute for paper labels. Japan is a notable pioneer: following amendment of the Pharmaceuticals and Medical Devices Act, paper-based PIs were abolished in principle for prescription drugs from August 2021 [28]. In contrast, extending e-labeling to over-the-counter medicines or nonhospital settings requires greater caution because patients may select and use these products without direct HCP supervision. A major gap in domain 1 was inconsistency in target labels, including the terminology used for product information and whether patient-facing and professional-facing information were distinguished. This definitional inconsistency may hinder international regulatory convergence. Several international stakeholder groups have proposed moving toward e-labeling implementation that could replace paper labels, cover all pharmaceuticals, and distinguish HCP- and patient-centered labels [14,15,17,18,29,30]. Before implementing e-labeling as the sole source of labeling information, jurisdictions should assess reliable internet access, access to digital devices, sufficient digital literacy, and a backup plan for users who cannot access electronic information. Therefore, uneven transition should not be interpreted as regulatory delay alone; rather, it reflects an attempt to balance the benefits of e-labeling with patient safety, digital literacy, and equity.

QR codes are currently the most common access mechanism, partly because users have become familiar with scanning QR codes. However, the specific access technology may be less important than the governance of the identifier and the reliability of the linked information. Some jurisdictions provided guidance on presenting URLs and user instructions with machine-readable codes and embedding standardized unique identifiers. Other issues, such as enabling batch-specific e-labels and using multifunctional codes rather than printing multiple codes on packages, were also addressed. A key implementation gap concerns the platform and formats used to provide e-labels. Platforms providing authority-approved labels were hosted by MAHs, regulatory authorities, or third parties. A transition pattern in digital format was also identified, moving from PDF to HTML and ultimately to structured, machine-readable formats. Structured formats are essential to support advanced functionalities and integration with other health care systems [1,2]. In particular, the EU ePI Common Standard, based on HL7 FHIR, provides a concrete example of how a common technical standard can support harmonized product information across centrally and nationally authorized medicines [31,32]. Future operational strategies should therefore prioritize structured, regulatory-approved product information provided through credible platforms. Responsibilities for each stage, from creating to providing e-labels, should also be clearly stated in legal frameworks.

Policies commonly referred to multilingual information, audio or video formats, assistive content, readability, font size, contrast, and searchability. These features are essential for realizing the added value of e-labeling beyond electronic access alone. Interoperability was most clearly addressed in the EU context, likely reflecting the early adoption of a common FHIR-based standard. The EU has considered the use of e-labeling not only in other health care systems, such as e-prescribing or adverse event reporting, but also in regulatory submission processes and information exchange across NCA systems [20,33]. Personalized information remains less mature, with policies providing only limited examples, such as saving user preferences and managing treatment information. This is understandable because themes in domain 3 introduce questions about privacy, data security, profiling, and the boundary between authorized information and interpreted or filtered information. The relative rarity of interoperability and personalization provisions may therefore reflect not only technical constraints but also the higher governance burden associated with data standards, workflow integration, version control, and privacy protection. Their presence was interpreted in this study as evidence of more advanced policy expansion rather than as a basic requirement for e-labeling adoption. Nevertheless, structured e-labeling creates the technical foundation for future expansions. Continued efforts are therefore needed to advance domain 3 maturity while ensuring the basic functionality of e-labeling.

These findings suggest that e-labeling regulation should evolve through a phased approach [34]. Early stages may focus on HCP-administered products, HCP-facing labels, or selected products with lower risk of use. In this phased framework, advanced functions such as interoperability and personalization should be approached as later-stage expansion goals rather than baseline requirements at the outset. As e-labeling expands toward more patient-centered use, later stages should consider broader coverage of human medicines, paper label replacement, integration with regulatory review and import or export processes, and user-level data handling. From a harmonization perspective, relevant multistakeholder groups should coordinate efforts ranging from a minimum common framework to broader legal alignment [34]. Such a framework could include standardized terminology, data elements, version identifiers, and structured data standards. ICMRA could support regulatory convergence by facilitating the exchange of pilot experiences, maturity assessment tools, and policy templates among regulators. HL7, ISO, GS1, and other standards organizations could contribute to technical alignment by supporting structured, machine-readable, interoperable data exchange mechanisms and internationally recognized identifiers [17,18,35]. WHO could frame e-labeling within national digital health strategies and equity-oriented digital health governance, while ITU, national governments, and telecommunications partners could support the infrastructure needed for meaningful digital access [14,34,36]. HCPs should be involved in user education, especially during the transition from paper to electronic information [15,18]. Usability testing and feedback from patient organizations will help establish an effective e-labeling implementation strategy. Public promotion campaigns may be needed to explain what e-labeling is, how to access it, and how to request alternatives when digital access is limited [18,34,36]. During this transition, paper label replacement should proceed only when users can reliably access and understand electronic information, with safeguards such as print-on-demand or other nondigital options remaining available for those who need them. A harmonized regulatory framework developed through phased implementation and collaboration with international standardization bodies would promote consistent information exchange, equitable access, and improved patient safety across digital health ecosystems [2,37,38].

The fsQCA findings were consistent with this interpretation. The analysis identified 1 conservative sufficient configuration associated with paper-label replacement. Policy maturity was central to this configuration. Its presence suggests that paper replacement is more likely when e-labeling has moved beyond a preliminary or supplementary stage and is embedded in a more established regulatory framework. Digital infrastructure readiness should be interpreted more cautiously because all ICMRA jurisdictions in the dataset had relatively well-developed digital environments. Given the limited cross-jurisdictional variation in digital infrastructure among the included ICMRA members, its high necessity consistency is better understood as a background condition within this sample rather than as a differentiating factor. The presence of explicit MAH responsibility highlights the need for a clearly assigned actor to maintain, update, and ensure access to e-labeling. Conversely, the absence of explicit NCA responsibility should not be interpreted as meaning that authorities withdraw from e-labeling governance. In this study, the MAH and NCA responsibility conditions were coded independently as binary conditions. Therefore, the absence of explicit NCA responsibility indicates that the policy documents did not assign direct operational responsibility to the authority, while regulatory oversight may still occur through approval and review.

### Comparison With Prior Work

Previous studies have examined the emergence and implementation of pharmaceutical e-labeling and broadly support the direction of our findings. Shaik et al [39] emphasized the need for harmonized e-labeling systems and positioned digital labeling as part of the broader digital health ecosystem, while identifying the lack of international standardization as a major barrier to implementation. Skogman-Lindqvist et al [40] provided empirical evidence from European hospital-based pilot programs, showing the practical value of electronic package leaflets while also noting implementation challenges in a regional context. Consistent with these studies, our findings indicate that e-labeling is increasingly viewed as a means of providing more timely, accessible, and interoperable product information, but that implementation remains uneven across jurisdictions. This study adds to prior work by examining e-labeling as a comparative regulatory policy issue across multiple ICMRA member jurisdictions. Rather than focusing on a single country, region, or pilot setting, we compared both legal and nonlegal policy instruments. We also examined differences in policy scope, implementation strategy, operational infrastructure, and advanced considerations. The results show that fragmentation is not only technical, but also regulatory and institutional. It is reflected in legal authority, medicine coverage, the relationship between electronic and paper-based information, responsibility allocation, and the extent to which interoperability or personalized information is addressed. To our knowledge, this is the first study to apply fsQCA to paper-label replacement policies in pharmaceutical e-labeling.

### Strengths

This study provides a comprehensive overview of current national approaches in the context of increased international attention to pharmaceutical e-labeling. The study has 3 main strengths. First, it assessed the e-labeling policies of ICMRA member jurisdictions using official national and regulatory documents. Both legally binding and nonlegal instruments were included to enable the analysis to capture the framing of e-labeling in practice and its positioning with respect to paper-based product information. Second, triangulation with international stakeholder documents helped clarify implementation status and interpret policy content where public regulatory information was uneven. This reduced the risk of confusing limited document availability with limited policy activity. Third, the study combined thematic analysis, maturity assessment and fsQCA into a single analytical structure. The qualitative and configurational analyses were closely integrated, as the maturity levels and fsQCA conditions were derived from the coded policy content. Conservative minimization without directional expectations provided a cautious basis for solution derivation. Moreover, the same sufficient configuration was retained under both alternative membership-score specifications in the calibration sensitivity analyses.

### Limitations

Despite these contributions, several limitations should be noted. First, the analysis was limited to ICMRA member jurisdictions. This scope was appropriate for examining e-labeling policy development among regulators engaged in international coordination, but it limits generalizability to countries outside this network, particularly lower-resource regulatory settings. Future studies should include a broader range of non-ICMRA jurisdictions to examine how e-labeling can be implemented equitably in settings with more limited digital access or regulatory capacity. Second, the analysis depended on publicly available policy documents. This may reflect documentation visibility rather than true policy absence. Jurisdictional comparisons should therefore be interpreted as comparisons of documented regulatory articulation, not necessarily the full extent of implementation activity. Nigeria and India were excluded because no eligible official e-labeling policy documents could be identified. This should not be interpreted as definitive evidence that no policy activity exists. For India, triangulation suggested fragmented e-labeling–related activity [18], whereas no relevant evidence was identified for Nigeria. These findings highlight the need for greater transparency in digital health regulation and for future studies that combine document analysis with regulator interviews or direct policy verification. Third, cross-jurisdictional comparison is inherently constrained by structural differences among regulatory systems. Differences in legal traditions, institutional arrangements, implementation capacity, and documentation practices may influence how e-labeling policies are written, implemented, and made publicly available. Accordingly, the maturity levels should not be interpreted as simple rankings of regulatory performance. Although the framework was informed by the logic of the WHO GBT, it was adapted for the specific context of pharmaceutical e-labeling and should be refined through further empirical validation. Fourth, the analysis involved interpretive judgment despite the use of independent coding, consensus procedures, and triangulation. Thematic coding, maturity classification, and fsQCA calibration required heterogeneous policy language to be translated into comparable categories, which is a common challenge in comparative qualitative policy analysis. Sensitivity analyses using alternative membership-score specifications yielded the same sufficient configuration, indicating that the configurational result was robust to the examined variations in calibration. Nevertheless, more substantial changes to the calibration scheme could potentially produce a different configuration. Because this study focused on ICMRA jurisdictions, variation in some contextual conditions was limited. The fsQCA findings should not be interpreted as causal estimates. Future research should examine how institutional, economic, and political factors shape e-labeling adoption through regulator interviews, implementation case studies, or established health policy implementation frameworks.

### Conclusions

This study reveals that pharmaceutical e-labeling has passed the early discussion phase in many ICMRA member jurisdictions, but its regulatory development is still unequal. Most policies have been directed at enabling electronic access to product information, while fewer have established the legal and operational basis needed to replace paper-based information. This difference was highlighted by the maturity assessment, which indicated that only a small group of jurisdictions have reached a more advanced stage of e-labeling regulation. The fsQCA further suggested that paper replacement is more likely to occur when e-labeling is accompanied by a mature policy framework and clear operational responsibility. Taken together, these findings underscore the need for jurisdictions to establish robust regulatory systems that enable reliable information updates, interoperability, and equitable access before transitioning from paper-based labeling to ePI.

Building on these findings, this study makes an innovative contribution by developing a regulatory maturity framework and the applying fsQCA to pharmaceutical paper-label replacement policies. Whereas previous studies have largely examined individual countries, regions, or pilot settings, this study systematically compares regulatory and implementation approaches across multiple ICMRA member jurisdictions. The identification of regulatory and operational conditions associated with paper-label replacement provides a structured basis for assessing regulatory readiness. In practice, the framework may assist regulators and policymakers in identifying priorities for legal and operational development, standards alignment, and implementation planning while accommodating jurisdiction-specific regulatory environments.

## Appendix

Multimedia Appendix 1 Additional tables.

## Abbreviations

AIFA: Italian Medicines Agency
ANSM: French National Agency for the Safety of Medicines and Health Products
APAC-Asia: Asia Partnership Conference of Pharmaceutical Associations
EFPIA: European Federation of Pharmaceutical Industries and Associations
e-labeling: electronic labeling
EMA: European Medicines Agency
ePI: electronic product information
EU: European Union
FDA: Food and Drug Administration
FHIR: Fast Healthcare Interoperability Resources
fsQCA: fuzzy-set qualitative comparative analysis
GBT: Global Benchmarking Tool
GS1: Global Standards 1
GSCF: Global Self-Care Federation
GTIN: Global Trade Item Number
HCP: health care professional
HL7: Health Level 7
ICMRA: International Coalition of Medicines Regulatory Authorities
IFPMA: International Federation of Pharmaceutical Manufacturers and Associations
ITU: International Telecommunication Union
MAH: marketing authorization holder
NCA: national competent authority
PI: package insert
PMDA: Pharmaceuticals and Medical Devices Agency
PRI: proportional reduction in inconsistency
PRISMA-S: Preferred Reporting Items for Systematic Reviews and Meta-Analyses literature search extension
QCA: qualitative comparative analysis
SPL: Structured Product Labeling
SRQR: Standards for Reporting Qualitative Research
WHO: World Health Organization

## Data Availability

The data supporting the findings of this study were obtained exclusively from publicly available regulatory and stakeholder documents. The included documents, source websites, search details, and inclusion/exclusion decisions are summarized in Tables S1 and S2 in Multimedia Appendix 1.
