# Supplementary material for: Regulatory Readiness for Paper Replacement in Global Pharmaceutical Electronic Labeling: Cross-Sectional Comparative Policy Analysis
Source: J Med Internet Res. 2026 Aug 18;28:e96787. doi: 10.2196/96787 (PMC13535302; doi:10.2196/96787)
Supplement: Supplementary file 1 [file jmir_v28i1e96787_app1.zip › Supplementary Table S3-6.docx]

**Supplementary Table S3. Coding frequency of identified e-labeling policy themes in the main and triangulation corpora**

Values indicate the number and percentage of eligible policy documents coded for each theme in the main policy corpus and triangulation corpus. A single document could be coded under more than one theme.

| Domain | Theme | Coded documents, n/N (%) | |
| --- | --- | --- | --- |
|  |  | **Main policy corpus**  **(N = 81)** | **Triangulation corpus**  **(N = 27)** |
| D1 | Target labels for e-labeling (Theme 1a) | 64/81 (79.0) | 15/27 (55.6) |
|  | Medicine categories (Theme 1b) | 66/81 (81.5) | 20/27 (74.1) |
|  | Transition approaches (Theme 1c) | 54/81 (66.7) | 24/27 (88.9) |
| D2 | Interface mechanisms (Theme 2a) | 59/81 (72.8) | 18/27 (66.7) |
|  | Entity providing e-label (Theme 2b) | 52/81 (64.2) | 20/27 (74.1) |
|  | Digital format (Theme 2c) | 24/81 (29.6) | 11/27 (40.7) |
| D3 | Accessibility and usability (Theme 3a) | 48/81 (59.3) | 18/27 (66.7) |
|  | Interoperability (Theme 3b) | 14/81 (17.3) | 14/27 (51.9) |
|  | Personalized information (Theme 3c) | 10/81 (12.3) | 7/27 (25.9) |
| ETC | Regulatory lifecycle efficiency and timely updates | N/A | 4/27 (14.8) |
|  | Implementation risks and transition safeguards | N/A | 5/27 (18.5) |

**Notes.** D1 = e-labeling scope and implementation strategy; D2 = operational infrastructure for e-labeling implementation; D3 = advanced considerations for e-labeling; ETC = other e-labeling-related content outside Domains 1–3; N/A = not applicable.

**Supplementary Table S4. Raw jurisdiction-level variables considered for fuzzy-set qualitative comparative analysis (fsQCA)**

| Country  /Region | Income^a^ | Digital infrastructure readiness^b^ | Personal digital-device adoption^c^ | Internet access / penetration rate^d^ | Number of first languages^e^ | Government digitalization^f^ |
| --- | --- | --- | --- | --- | --- | --- |
| Brazil | Upper-middle | 82.00 | 87.38% | 84.46% | 1 | VHEGDI |
| Mexico | Upper-middle | 80.70 | 81.21% | 83.12% | 1 | HEGDI |
| United States | High | 96.70 | 97.05% | 94.69% | 1 | VHEGDI |
| Canada | High | 88.60 | 95.68% | 94.35% | 2 | VHEGDI |
| Republic of South Africa | Upper-middle | 83.60 | 86.97% | 78.36% | 2 | HEGDI |
| China | Upper-middle | 85.80 | 93.51% | 92.00% | 1 | VHEGDI |
| Japan | High | 93.20 | 93.78% | 85.54% | 1 | VHEGDI |
| Republic of Korea | High | 94.40 | 97.89% | 97.90% | 1 | VHEGDI |
| Singapore | High | 97.80 | 99.80% | 94.38% | 2 | VHEGDI |
| United Kingdom | High | 93.60 | 95.92% | 95.47% | 1 | VHEGDI |
| France | High | 89.80 | 95.86% | 88.65% | 1 | VHEGDI |
| Germany | High | 87.80 | 91.33% | 93.50% | 1 | VHEGDI |
| Italy | High | 87.70 | 95.95% | 89.22% | 1 | VHEGDI |
| Netherlands | High | 92.50 | 91.64% | 97.01% | 1 | VHEGDI |
| Ireland | High | 90.70 | 97.83% | 97.19% | 2 | VHEGDI |
| Sweden | High | 95.30 | 95.10% | 95.53% | 1 | VHEGDI |
| Switzerland | High | 92.40 | 98.51% | 97.34% | 2 | VHEGDI |
| Australia | High | 95.10 | 97.49% | 96.13% | 1 | VHEGDI |
| New Zealand | High | 90.30 | 95.90% | 93.52% | 2 | VHEGDI |

^a^ World Bank Country and Lending Groups, FY2026.

^b^ ITU ICT Development Index 2025, Table 1. The source note in the working dataset identifies the measure as the IDI score.

^c^ ITU DataHub, Individuals who own a mobile (%), 2024.

^d^ ITU DataHub, Individuals using the Internet (%), 2024.

^e^ CIA World Factbook 2024, Languages.

^f^ United Nations E-Government Development Index (EGDI) Data Center; VHEGDI = very high EGDI; HEGDI = high EGDI

**Supplementary Table S5. Necessity analysis for paper-label replacement**

| Condition | Consistency | Coverage | Relevance of necessity |
| --- | --- | --- | --- |
| Digital infrastructure readiness | 0.980 | 0.521 | 0.229 |
| E-labeling policy scope | 0.655 | 0.457 | 0.477 |
| E-labeling policy maturity | 0.944 | 0.723 | 0.700 |
| Explicit marketing authorization holder responsibility | 0.678 | 0.500 | 0.538 |
| Explicit national competent authority responsibility | 0.429 | 0.380 | 0.592 |
| Absence of digital infrastructure readiness | 0.253 | 0.950 | 0.993 |
| Absence of e-labeling policy scope | 0.644 | 0.905 | 0.955 |
| Absence of e-labeling policy maturity | 0.458 | 0.544 | 0.773 |
| Absence of explicit marketing authorization holder responsibility | 0.322 | 0.407 | 0.743 |
| Absence of explicit national competent authority responsibility | 0.571 | 0.561 | 0.717 |

**Supplementary Table S6. Truth table for sufficiency analysis of paper-label replacement**

| Digital infrastructure | Policy scope | Policy maturity | MAH responsibility | NCA responsibility | OUT | n | Inclusion | PRI | Cases |
| --- | --- | --- | --- | --- | --- | --- | --- | --- | --- |
| 1 | 0 | 1 | 1 | 0 | 1 | 5 | 0.959 | 0.941 | Australia; Switzerland; Republic of Korea; Brazil; China |
| 1 | 1 | 1 | 1 | 0 | 1 | 2 | 0.880 | 0.667 | Canada; Singapore |
| 1 | 1 | 1 | 0 | 1 | 0 | 5 | 0.679 | 0.514 | Japan; France; South Africa; Sweden; Italy |
| 1 | 1 | 1 | 1 | 1 | 0 | 3 | 0.499 | <0.001 | New Zealand; Netherlands; Germany |
| 1 | 1 | 0 | 1 | 1 | 0 | 2 | 0.457 | <0.001 | United States; Ireland |
| 1 | 1 | 0 | 0 | 0 | 0 | 2 | 0.222 | <0.001 | Mexico; United Kingdom |

**Notes.** Truth-table settings: inclusion cutoff = 0.80; frequency cutoff = 2 cases per configuration. For fuzzy-set conditions, 1 and 0 indicate whether the case membership was above or below the crossover point for the purpose of truth-table construction; inclusion and PRI were calculated using fuzzy-set membership scores. OUT is the truth-table output assigned to the outcome set. Only observed configurations meeting the frequency cutoff are shown.

**Abbreviations:** MAH, marketing authorization holder; NCA, national competent authority; OUT, output value; n, number of cases in the configuration; PRI, proportional reduction in inconsistency.
